# Deep Machine Learning for Oral Cancer: From Precise Diagnosis to Precision Medicine

**DOI:** 10.3389/froh.2021.794248

**Published:** 2022-01-11

**Authors:** Rasheed Omobolaji Alabi, Alhadi Almangush, Mohammed Elmusrati, Antti A. Mäkitie

**Affiliations:** ^1^Research Program in Systems Oncology, Faculty of Medicine, University of Helsinki, Helsinki, Finland; ^2^Department of Industrial Digitalization, School of Technology and Innovations, University of Vaasa, Vaasa, Finland; ^3^Department of Pathology, University of Helsinki, Helsinki, Finland; ^4^Institute of Biomedicine, Pathology, University of Turku, Turku, Finland; ^5^Department of Otorhinolaryngology – Head and Neck Surgery, University of Helsinki and Helsinki University Hospital, Helsinki, Finland; ^6^Division of Ear, Nose and Throat Diseases, Department of Clinical Sciences, Intervention and Technology, Karolinska Institute and Karolinska University Hospital, Stockholm, Sweden

**Keywords:** machine learning, deep learning, oral cancer, prognostication, precision medicine, precise surgery

## Abstract

Oral squamous cell carcinoma (OSCC) is one of the most prevalent cancers worldwide and its incidence is on the rise in many populations. The high incidence rate, late diagnosis, and improper treatment planning still form a significant concern. Diagnosis at an early-stage is important for better prognosis, treatment, and survival. Despite the recent improvement in the understanding of the molecular mechanisms, late diagnosis and approach toward precision medicine for OSCC patients remain a challenge. To enhance precision medicine, deep machine learning technique has been touted to enhance early detection, and consequently to reduce cancer-specific mortality and morbidity. This technique has been reported to have made a significant progress in data extraction and analysis of vital information in medical imaging in recent years. Therefore, it has the potential to assist in the early-stage detection of oral squamous cell carcinoma. Furthermore, automated image analysis can assist pathologists and clinicians to make an informed decision regarding cancer patients. This article discusses the technical knowledge and algorithms of deep learning for OSCC. It examines the application of deep learning technology in cancer detection, image classification, segmentation and synthesis, and treatment planning. Finally, we discuss how this technique can assist in precision medicine and the future perspective of deep learning technology in oral squamous cell carcinoma.

## Introduction

Oral squamous cell carcinoma (OSCC) is a common cancer that has an increased incidence across the globe [1–3]. Traditionally, the preferred primary cornerstone therapy for OSCC is surgical treatment [4]. Additionally, considering the aggressive nature of OSCC and the fact that many patients are diagnosed with locoregionally advanced disease, multimodality therapy with concomitant chemoradiotherapy becomes imperative [1]. In spite of the afore-mentioned treatment possibilities, the high incidence rate and the suboptimal treatment outcome still form a significant concern.

Early-stage diagnosis is of utmost importance for better prognosis, treatment, and survival [5, 6]. This is important to enhance the proper management of cancer. Late diagnosis has hampered the quest for precision medicine despite the recent improvement in the understanding of the molecular mechanisms of cancer. Therefore, deep machine learning technique has been touted to enhance early detection, and consequently to reduce cancer-specific mortality and morbidity [7]. Automated image analysis clearly has the potential to assist pathologists and clinicians in the early-stage detection of OSCC and in making informed decisions regarding cancer management.

This article discusses the overview of deep learning for OSCC. It examines the application of deep learning for precision medicine through accurate cancer detection, effective image classification, and efficient treatment planning for proper management of cancer. Finally, we discuss the prospect of future development of the deep learning-based predictive model for use in the daily clinical practice for improved patient care and outcome.

In this study, we performed a systematic review of the published systematic reviews that have examined the application of deep learning in oral cancer. To the best of our knowledge, this is the first systematic review of the published systematic reviews on the application of deep learning for oral cancer prognostication. This research approach provides the opportunity to offer a summary of evidence to clinicians regarding a particular domain or phenomena of interest [8, 9].

We have decided to use this approach of a systematic review of systematic reviews because it offers the opportunity to summarize the evidence from more than one systematic review that have examined the application of deep learning for prognostication oral cancer outcomes. In our previous study, we examined the utilization of deep learning techniques for cancer prognostication [7]. In this present study, we aim to summarize the evidence relating to the qualitative (reliability of the studies that examined deep learning for cancer prognostication) and quantitative (performance metrics of the deep learning algorithms) parameters that are needed to provide insightful overview on how deep learning techniques have helped in the improved and effective management of oral cancer. This will contribute to the effort to develop adequate cancer management by providing elements for the process from precise diagnosis to precise medicine.

## Materials and Methods

The research question was: “How has deep learning techniques helped in achieving precise diagnosis or prognostication of oral cancer outcomes in the quest for precise medicine?”

### Search Strategy

**Figure 1:**
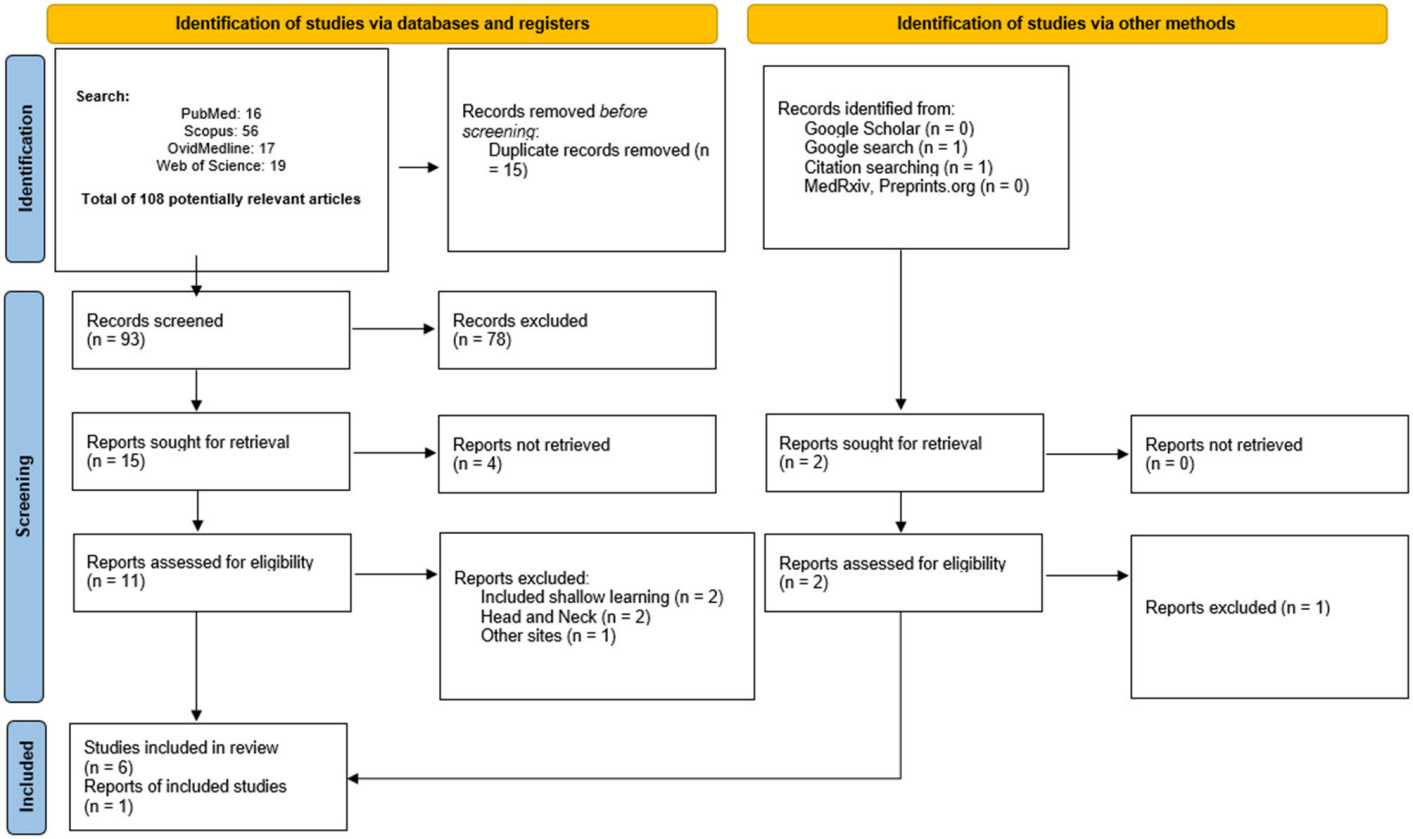
A simplified illustration of a deep learning algorithm architecture with an input data ([9], figure modified).

Detailed automated literature searches were performed in PubMed, OvidMedline, Scopus, and Web of Science from inception until the end of October 2021. Additionally, Google scholar, and unpublished studies were searched for possible gray literatures. This helps to reduce research waste. Similarly, the reference lists of potentially relevant systematic reviews were searched to ensure that all potential systematic reviews have been included. The manual searching of the reference list of the potential systematic reviews helps to avoid selection bias. The RefWorks software was used to manage the relevant systematic reviews and remove any duplicate reviews. The updated Preferred Reporting Items for Systematic Review and Meta-Analysis (PRISMA) was used in the searching and screening processes (Figure 1).

### Search Protocol

The search protocol was developed by combining search keywords: [(“deep learning AND oral cancer AND review”)]. The retrieved hits were exported to RefWorks software for further analysis.

### Inclusion Criteria

All systematic reviews that considered deep learning for oral cancer diagnosis and prognosis were included. This includes reviews and scoping reviews that examined the application of deep learning on oral cancer prognostication.

### Exclusion Criteria

Considering the fact that this study is a systematic review of systematic reviews, original articles, systematic reviews of other cancer types, comments, opinions, perspectives, editorials, abstracts only, and articles in languages other than English were excluded (Figure 1). This exclusion criteria was important as this study aims to put into the right perspectives, everything that has been summarized in the systematic review papers in other to answer the focused question presented in this study. Similarly, systematic reviews relating to head and neck cancer were excluded. Considering the vast amount of published studies on neural networks [10–15], we have excluded details about neural network and primarily focused on the role of deep learning techniques in the quest for precision medicine.

### Quality Appraisal

The quality appraisal and risk of bias of the included systematic reviews ensure that the quality of the included systematic reviews is reliable. All potentially relevant systematic reviews were subjected to quality guidelines for systematic review as recommended by the National Institute of Health Quality Assessment tools [16]. The included studies in this review were subjected to four quality criteria informed by the same quality assessment and risk of bias tool [17]. These criteria were modified to include design (review or systematic review), methodology (databases were systematically searched), interventions (deep learning methodology was applied and performance measures from the deep learning algorithms was presented), and statistical analysis (conclusion from the included studies). These criteria have been scaled such that score 0 for “NO” and “Unclear” and score 1 for “Yes” for each of these criteria. Thus, a total of 4 points is expected from these criteria. Scaling of these criteria to percentage means that it was based on a 100% scale (i.e., 25% for each criterion). Thus, a potentially relevant systematic review or review with a quality score (≥50%) were considered reasonable and qualified to be included in this systematic review of systematic reviews (Table 1).

**Table 1:** The quality appraisal of the included systematic reviews.

**Study**	**Design**	**Methodology**	**Interventions**	**Statistical analysis**	**Score (%)**	**Interpretation**
Nagi et al. [18] (India)	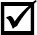	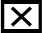	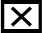	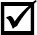	50	Medium
Panigrahi and Swarnkar [19] (India)	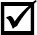	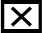	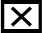	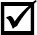	50	Medium
Sultan et al. [20] (United States)	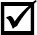	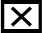	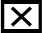	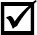	50	Medium
Alabi et al. [7] (Finland)	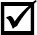	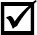	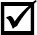	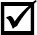	100	High
Ren et al. [21] (China)	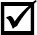	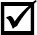	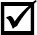	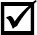	100	High
Chu et al. [22] (China)	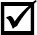	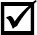	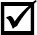	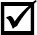	100	High
García-Pola et al. [23] (Spain)	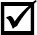	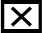	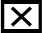	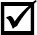	50	Medium

### Extraction From Eligible Systematic Review

In each eligible systematic review, the first author name, year of publication, country, the title of studies, number of included studies, and conclusions derived from the systematic review of the application of deep learning for prognostication were summarized (Table 2). The detailed explanation of the application of deep learning that that had transformed from precise diagnosis to precise medicine was discussed collectively in the discussion section.

**Table 2:** Summary of review studies on the application of deep learning in oral cancer.

**Review (Country)**	**Topic**	**Number of studies included**	**Performance reported**	**Conclusion**
Nagi et al. [18] (India)	Clinical applications and performance of intelligent systems in dental and maxillofacial radiology: A review	10	NR	Intelligent systems such as deep learning have been proven to assist in making clinical diagnostics recommendation and treatment planning
Panigrahi and Swarnkar [19] (India)	Machine learning techniques used for the histopathological image analysis of oral cancer—A review	8 14 (deep learning with histopathological images)	NR	Computer-aided approach such as machine learning can assist in the prediction and prognosis of cancer
Sultan et al. [20] (United States)	The use of artificial intelligence, machine learning and deep learning in oncologic histopathology	9 (histopathological images)	NR	Artificial intelligence has the potential for personalizing cancer care. Furthermore, it can achieve excellent and sometimes better results than the human pathologists. The AI model should provide a second opinion to the expert pathologists to reduce potential diagnostic errors
Alabi et al., [7] (Finland)	Utilizing deep machine learning for prognostication of oral squamous cell carcinoma—A systematic review	34	Average accuracy was 81.8% for computed tomography images Average accuracy was 96.6% for spectra data	Deep learning approach offers a promising potential in the prognostication of oral cancer
Ren et al. [21] (China)	Machine learning in dental, oral and craniofacial imaging: a review of recent progress	27	NR	The application of deep learning approach for image detection has been intense in the past few years. Meanwhile, certain areas need to be supplemented for sustainable deep-learning research application in oral and maxillofacial radiology
Chu et al. [22] (China)	Deep learning for clinical image analyses in oral squamous cell carcinoma—A review	14	Range from 77.89 to 97.51% (pathological images) Range from 76 to 94.2% (radiographic images)	The trained deep learning model has the potential of producing a high clinical translatability in the proper management of oral cancer patients
García-Pola et al. [23] (Spain)	Role of artificial intelligence in the early diagnosis of oral cancer. A scoping review	36		Artificial intelligence can help in the detection of oral premalignant disorder. In addition, it can help in the early diagnosis of oral cancer Artificial intelligence can help in the precise diagnosis and prognosis of oral cancer

## Results

### Characteristics of Relevant Studies

All the included systematic reviews were conducted in English language. Of the 7 included systematic reviews [7, 18–23], 4 were conducted in Asia (i.e., 2 each from China and India) [18, 19, 21, 22] while 2 reviews was conducted in Europe (Finland) [7, 23] and one review in the United States [20] (Table 2). The quality appraisal for all the review papers considered in this study indicated that 42.9% showed high quality [7, 21, 22] while 57.1% showed medium quality [18–20, 23]. Three of the reviews were conducted in the year 2020 [18–20] while four were conducted in the year 2021 [7, 21–23].

### Data Used in Deep Learning Analyses

The findings of the published reviews (summarized in Table 2) suggested that the application of deep learning in oral cancer management (diagnosis and prognosis) is heightening based on the number of studies included in the considered reviews. Several data types such as pathologic and radiographic images have been used by deep learning in the quest to achieve precise diagnosis and prognosis [20, 22–32]. Other data types include gene expression data, spectra data, saliva metabolites, auto-fluorescence, cytology-image, computed tomography images, and clinicopathologic images that have been used in the deep learning analysis for improved diagnosis of oral cancer [7].

### Performance of the Deep Learning Models in Oral Cancer Prognostication

The average reported accuracies were 96.6 and 81.8% for spectra data and computed tomography, respectively [7]. In addition, the accuracies ranged from 77.89 to 97.51% for pathological images and 76.0 and 94.2% for radiographic images [22]. This reveals that deep learning models have the potential to assist in the precise diagnosis and prognosis of oral cancer. These models carry the potential of being useful tools for clinical practice in oral oncology.

Remarkably, it is hoped that the use of disruptive technology approach such as deep learning techniques would address some of the concerns of the traditional diagnostic methods. For instance, it is sometimes challenging to differentiate between malignant and non-malignant adjacent tissues. This makes it challenging for the surgeons and oncologists to either properly detect and localize tumors before a biopsy, determine the surgical resection margins, or properly evaluate the resection bed after tumor extraction during post-treatment follow-up by for example endoscopy [33]. As a result, various imaging modalities are routinely used as diagnostic tools in oncology. In spite of this, this approach is subjective as the experience of the radiologists will play an important role in obtaining accurate diagnosis [33].

Of note, histopathological approach (biopsy) is the gold standard for oral squamous cell carcinoma. However, obtaining histopathological images is time-consuming, invasive in nature, subjective to pathologists' judgement, and relatively expensive. Interestingly, hyperspectral imaging techniques offer a potential non-invasive approach for tumor detection and assessment of pathological tissue by illustrating the spectral features of different tissues [21, 33, 34]. With this approach, data can become easily available, which can help the low-volume centers to acquire experience of high-volume centers for the patient's benefit.

Despite these benefits from using hyperspectral imaging techniques, several implications should be considered. One of the possible implications of this is that clinical examinations using these techniques might need more resources due to the needed learning phase and costs. In addition, drawing conclusions for treatment decisions and prognostic purposes based on subjective information from these methods, and not from histopathological diagnosis, has raised a valid concern [33]. Therefore, it has been suggested that each patient's finding should be images multiple times to obtain evidence of reproducibility of the image and subsequently, reliability of the diagnosis [33]. Similarly, injecting a fluorescent material (porphyrin) which can be potentially hazardous has raised another issue worthy of consideration [33]. Nonetheless, a laser-induced fluorescent technique have been proposed to overcome this concern [33, 35, 36]. Irrespective of the data type used in the training of the deep learning-based prognostication model, it is important to emphasize that the judgement of the clinicians still forms the bedrock of the decision-making. These models are poised to serve as an assistive tool to aid clinical decision-making.

## Discussion

### Deep Learning in Oral Cancer Management: An Overview

The advancements in technology in terms of increased computational power have allowed for improvement in model architecture of shallow machine learning to deep machine learning [7, 37, 38]. Owing to the fact that deep learning is a modification to the traditional neural network, it is also referred to as the deep neural network (DNN) [37]. Considering the continuous generation of data by various medical devices and digital record systems, it is important to process and manipulate these data accurately to obtain meaningful insights from them.

**Figure 2:**
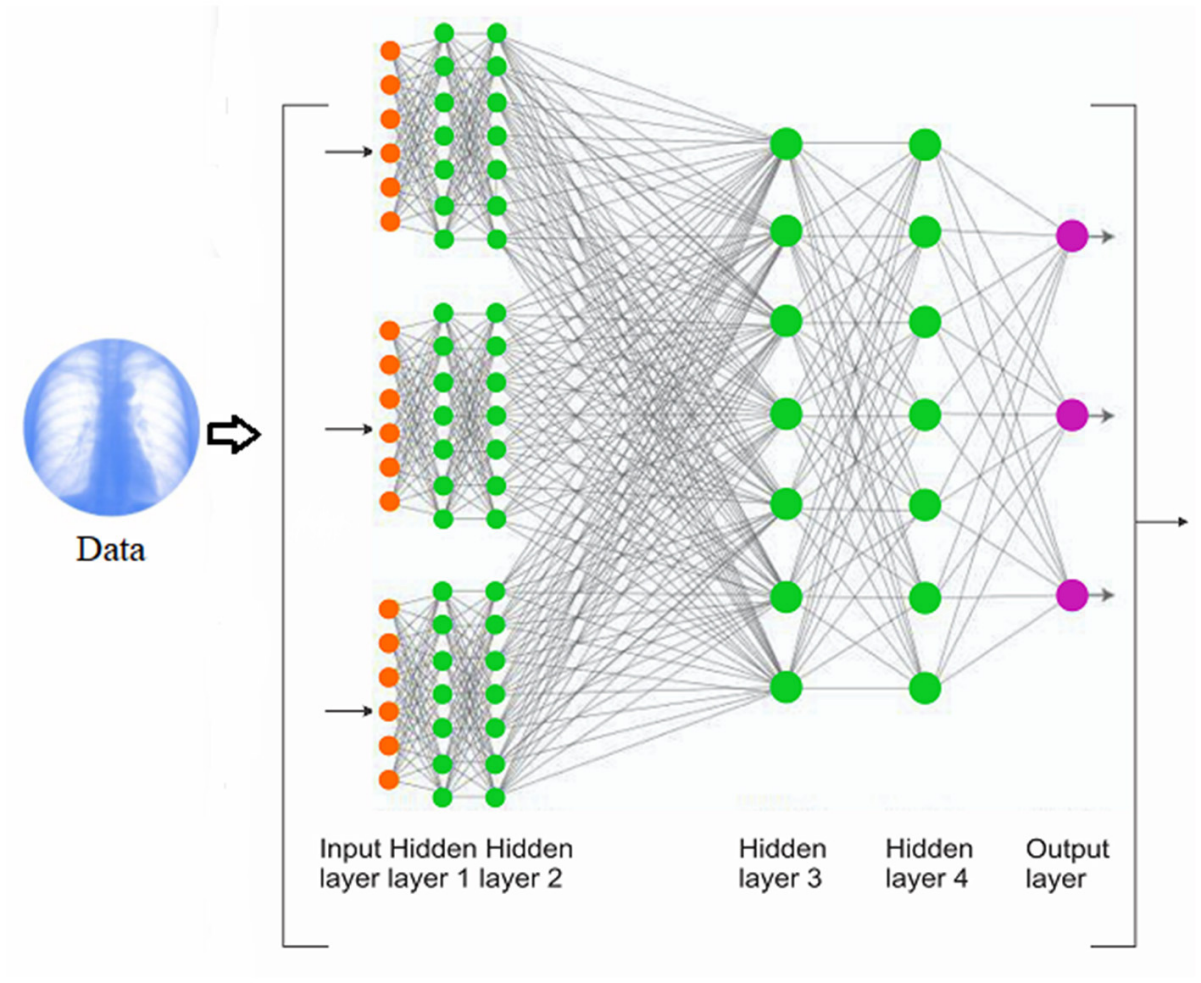
The schematic pipeline of the training process of a convolutional neural network.

Deep learning approach offers an insightful approach to support clinical decision-making. It is unique in the sense that it is able to devise its own representation that is needed for pattern recognition [38]. Typically, deep learning is made up of multiple layers that are arranged sequentially with a significant amount of primitive, non-linear operations such that the input fed into the first layer goes to the second layer and to the next layer (transformed into more abstract representations) until the input space is iteratively transformed into a distinguishable data points [38, 39] (Figure 2).

In general, deep learning may take any form of the learning paradigms. The motive for the application of deep learning determines the learning approach. In some cases, these learning paradigms can be mixed to achieve good model performance. For example, the deep neural network (DNN) was initially trained based on unsupervised learning paradigm during the learning phase. This was followed by supervised fine-tuning of stacked network [40–42]. The stacked auto-encoders (SAEs) and deep belief network (DBN) were examples of architectures that exhibited this approach. However, this approach requires complex techniques and significant amount of engineering to achieve good performance. Therefore, the supervised method became the most popularly used since it simplified the entire training process [40]. The convolutional neural network (CNN) and the recurrent neural network (RNNs) are examples of the architectures that have used the supervised learning paradigm [40]. To this end, most of the examined architectures used in the published studies were based on CNN or its derivatives. Although, two unsupervised architectures—variational auto-encoder (VAE) and generative adversarial network (GAN) are beginning to attract attention. It would be interesting to explore their applications in the review of medical images [40].

In the deep learning approach which is based on the supervised learning paradigm, the dataset is made up of input data points and corresponding output data labels [38, 40, 43]. Similarly, meaningful information is extracted from the data to enhance the delineation of features of clinical interest and importance (segmentation) [40, 43]. Of note, deep learning can featurize and learn from a variety of data types such as image, temporal, and multimodal inputs [38].

In medicine, especially in cancer management, primary diagnostic imaging modalities are fed into deep learning to achieve a trained model that can offer effective prognostication of cancer outcomes [44]. Examples of these highly dimensioned mineable primary diagnostic imaging modalities include radiomic data, ultrasound (US), computed tomography (CT), genomic, magnetic resonance imaging (MRI), and positron emission tomography (PET) [44]. Similarly, stained tissue sections from biopsies or surgical resections from high-resolution whole-slide images produce cytological and immunohistochemical data which have been used as inputs for the deep learning techniques.

Other non-invasive tools/data types include infrared thermal imaging, confocal laser endomicroscopy, multispectral narrow-band, and Raman spectroscopy [44]. From these different types, it has been reported that the computed tomography (or enhanced computed tomography) provides the widely used non-invasive data type for deep learning analysis [7]. Similarly, spectra data were reported as a form of an emerging non-invasive data type for precision and personalized cancer management using deep learning techniques [7, 44].

### Convolutional Neural Network

A typical CNN is made up of input and output layers alongside other important layers such as convolution, max pooling, and fully connected layers. These layers are essential to facilitate effective learning of more and more abstract features from the input data such as an image. When the input is fed into a typical CNN, the convolution layer takes the input image and extracts a feature. The extracted feature is transferred into the pooling layers for further processing. Finally, the reduced input images are then fed into the fully connected layer to classify the images into the corresponding labels. The entire process of building a deep learning-based prognostic model is summarized below (sub-section Inclusion Criteria). The CNN is an important type of neural network that has found application in medicine due to the fact it has addressed the various disadvantages of the traditional fully connected layers such as computational overheads. Several types have CNNs have evolved over time such as LeNet, AlexNet, DenseNet, GoogLeNet, ResNet, VGGNet [22]. However, the principle of operations of these variants remains largely the same.

### Pipeline for Building a Deep Learning-Based Prognostic Model

**Figure 3:**
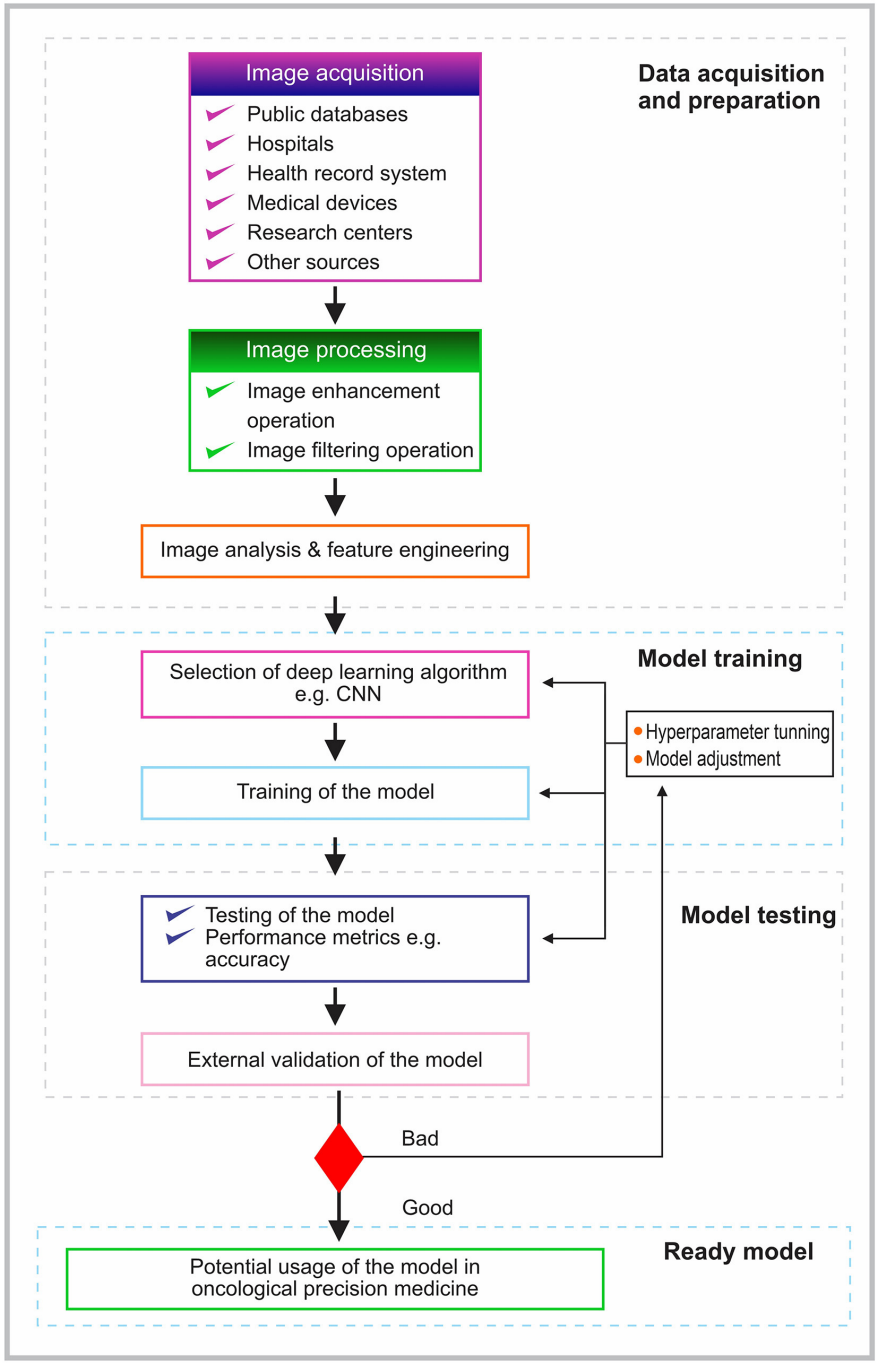
A schematic of a typical deep machine learning training process.

The typical pipeline for building a deep learning model involves image acquisition, preprocessing, image analysis, and feature engineering [21] (Figure 3). In image acquisition, the image type to be used in the analysis is acquired from the chosen source. The acquired images are preprocessed to remove artifacts and noise [21]. Of note, it is important to properly pre-process the data as the quality of data provided as inputs will determine the quality of results [19, 22, 45]. Therefore, using high-quality images is essential in the new era of precision medicine.

Several image enhancements and filtering options may be applied to improve the quality of the image [46] (Figure 3). For example, attempts such as the use of content-aware image restoration and Noise2void are deep learning methods being used in the attempt to denoise and restore bioimages in the era of deep learning [47–50]. Following the preprocessing step is the analysis and feature engineering stage. At this stage, necessary and required features are extracted. Considering the size of high-quality image data, it has been suggested to use certain pre-extracted features or to downsize the image sizes. There are two major drawbacks with these suggestions. Firstly, there is no confirmation which parts of the image have the most important information for proper diagnosis or prognosis. The image may contain some points that may have been inadvertently downsized. Secondly, the manual identification of important features (pre-extracted) on the image by the professionals may miss out on other extremely important features for precise diagnosis and prognosis. As a result, the detection of the regions of interest is done automatically by using a detection network (segmentation) [25]. The preprocessed image data are subsequently used for training (Figure 3).

The preprocessed data are usually divided into the training and testing sets, respectively [5, 51]. The training set, that is, the preprocessed data with outcome annotations are fed into a machine learning algorithm such as the Convolutional Neural Network (CNN) to perform the required tasks. A typical CNN is made up of convolutional, pooling, and fully connected layers. The processed training set is fed into the network through the convolutional layer. This layer automatically performs the image feature extraction to produce a feature map [21, 22, 52–55]. However, the produced feature map is still large and needs to be downsized [21]. An intuitive approach produced to downsize the feature map is the pooling process which works in a similar way as the earlier described convolution operation but with a different purpose (i.e., to downsize the feature map) [21, 54]. To this end, the filter used in the pooling process is designed to either generate the maximum value or average value. Thus, two types of pooling process is involved in the pooling layer (max-pooling or mean-pooling) [21]. Of note, there are concerns of excessive loss of information and possible destruction of partial information in processed images with the pooling process [21]. Although, several attempts have been made to address these concerns [56–59].

Thus, the output from the convolution/max pooling process is flattened into a single vector of value to facilitate processing and statistical analysis in the next phase (feeding to the fully connected layers) [52, 60]. The result from the afore-mentioned process (convolution/max pooling) is then fed into the fully connected layer to classify the images into the corresponding labels. Typically, a fully connected layer is made up of input, hidden, and output layers [22]. It connects all neurons in one layer to all the neurons in another layer. Hence, a linear transformation is used to combine inputs from the neurons in the previous layer to the next layer so that a single signal is formed which is then output to the next layer through an activation function to produce a non-linear transformation [21, 52, 61]. The most widely accepted activation function used for this purpose is the rectified linear unit (Relu) for two-class classification tasks and the softmax function typically for multi-class classification problems [52, 53, 55].

Based on the generated expected output, there may be a need for hyperparameter tuning and model adjustment to maximize the overall predictive performance of the model [22]. The model with suitable performance can be tested with the test sets and externally validated to ensure that the model works as expected (Figure 3). In general, the process of feature extraction and classification using a deep learning approach is carried out in a forward and backward manner in all the corresponding layers (convolution, pooling, and fully connected layers, respectively) [22].

## Deep Learning For Oral Cancer: From Precise Diagnosis To Precision Medicine

Precision medicine considers numerous technologies for designing and optimizing the route for personalized diagnosis, prognosis, and treatment of patients [62]. Precise diagnosis and prognosis of oral cancer are of utmost importance for treatment-related decision making in precision medicine. The realization of this offers the potential to improve patient care and outcome by guiding the clinicians toward carefully tailored interventions [22]. Additionally, it provides the opportunity to shift decisions away from trial and error and to reduce disease-associated health burdens [63].

The increased computing performance and the availability of large medical datasets (such as cancer datasets) in recent years are essential to the implementation of precision medicine. This is because technologies such as deep learning are capable of using large multidimensional biological datasets that capture individual variabilities in clinical, pathologic, genomic, biomarker, functional, or environmental factors [62, 63]. The approach is able to identify hard to discern patterns in these datasets that might not be easily discoverable by the traditional statistical approach [51, 63]. Thus, this methodology has shown significant accuracy in the precise diagnosis and prognosis of oral cancer [7].

For example, for precise diagnosis purposes, deep learning models have been used in the detection of oral cancer [24, 25, 64–75]. Additionally, these models have assisted in the prediction of lymph node metastasis [27–29, 76]. Besides, they have been reported to perform well in differentiating between precancerous and cancerous lesions [64, 77–81]. These models have been integrated to offer an automated diagnosis of oral cancer [24, 64, 82]. This approach offers the opportunity for cost-efficient early detection of oral cancer [64, 69, 70, 73, 80, 82], which is the basis for the development of management of oral cancer [21]. Proper management of oral cancer will subsequently lead to an increase in the survival of patients through improved treatment planning by providing precision and personalized medicine [5, 21].

Similarly, deep learning models have shown promising value in the prognostication of outcomes in oral cancer. For instance, deep learning models have been reported to show significant performance in the quantification of tumor-infiltrating lymphocytes [30], multi-class grading for oral cancer patients [26, 83], predicting disease-free survival in oral cancer patients [32], and dividing patients into risk groups and identifying patient groups who are at a high risk of poor prognosis [84].

Therefore, deep learning methodology can assist to achieve more precise detection, diagnosis, and prognostication in oral cancer management. The quest for precision medicine is poised to ensure that oral cancer is detected early, the cost of tests and mistakes in the recognition procedure can be reduced [19]. These advantages can translate into an improved chance of survival and better management of oral cancer [19, 21]. The workload of the clinicians can be greatly reduced since the data can be rapidly processed to obtain important prognostication of patients outcomes [18]. The cognitive bias and mistakes associated with treatment planning for patients can be reduced with automated deep learning tools to predict treatment outcomes [18].

In conclusion, based on the aforesaid, high-performance computing capabilities and the availability of large biological datasets of cancer patients are essential to achieve precision medicine. At the center of this high computational power are deep learning models that are able to identify patterns in multidimensional datasets.

## Challenges Of Deep Learning In Precision Medicine

Despite the reported potential benefits of deep learning techniques toward precision medicine, several important challenges must be resolved to achieve these. Firstly, quality of data (such as pathological or imaging) to be used in the analysis must be significantly high. To achieve this, a standardized resolution for the images should be agreed upon between for example the machine learning experts and the pathologists. This will ensure that the resolution is sufficient enough to capture histological features of the tumor and its microenvironment [22]. There should be a standardized guideline for both pathological and radiological image acquisition to obtain images that are beneficial in the deep learning analysis and ultimately, for precision medicine purposes.

Deep learning has better performance i.e., higher accuracy than the traditional shallow machine learning method but it is also more complex and this increases concern of explainability and interpretability of the model. There is a valid concern of the possible tradeoff between accuracy, explainability, and interpretability and this poses a challenge [85, 86].

It is important that the developed models are generalizable. Therefore, the model should be trained with a large dataset of high image quality. Another way to achieve a generalizable model is to manipulate the real data to obtain properly varied data for the training of deep learning [87]. The generalizability of the model ensures that the model provides a satisfactory performance measure within the entire intended target population. While external validation may ensure that the model will work as intended and without bias, it is important that the model shows good performance on a differing population regarding some properties [87]. Interestingly, research efforts are now ongoing to develop a generalizable model [87].

### Future Consideration

The arguments for enhancing precision medicine i.e., offering better individualized prognostication and management of oral cancer are important for improved overall outcomes. In spite of this, it is important to mention that the application of deep learning models to aid in introducing precision is still in its early stage of development. Several factors still need to be considered as mentioned above. For instance, the deep learning approach should be standardized to improve data preprocessing, standardized reporting of deep learning methodologies, and performance metrics of the corresponding models.

Additionally, these models are externally validated with different cohorts to ensure that the accuracy of the models is as reported. In fact, the accuracy of the developed model should be reported based on the performance of the model on external cohorts (external validation) to get its true performance [88]. These models should perform better than the human experts to fulfill the touted benefit as a clinical support system. Concerted efforts should be made to develop powerful AI algorithms that can handle available clinical dataset while producing acceptable prognostication performance. Moreover, necessary frameworks should be developed to guide the adoption of these models in daily clinical practices. The failure to consider all these may lead to accumulated number of studies on the potentials of deep learning methodology in the proper prognostication of patient outcome but without a true benefit to an individual oral cancer patient. Similarly, this is warranted before their use in daily clinical practice as a clinical support system to provide a second opinion to the clinician.

Apart from the aforementioned, continuous efforts should be made to increase the availability of the existing multidimensional biological and clinical data for deep learning approaches. This is important to further increase the volume of datasets and thus, the benefits of the application of deep learning, which seems to be a promising new tool for precision medicine of oral cancer.

## Data Availability Statement

The original contributions presented in the study are included in the article/supplementary material, further inquiries can be directed to the corresponding author/s.

## Author Contributions

RA and AM: study concepts and study design. RA: studies extraction. AM and AA: acquisition and quality control of included studies. RA, ME, AA, and AM: data analysis and interpretation. RA and AA: manuscript preparation. AM and ME: manuscript review. AA and RA: manuscript editing. All authors approved the final manuscript for submission.

## Funding

This work was funded by Helsinki University Hospital Research Fund (No. TYH2020232) and Sigrid Jusélius Foundation (No. Antti Mäkitie 2020).

## Conflict of Interest

The authors declare that the research was conducted in the absence of any commercial or financial relationships that could be construed as a potential conflict of interest.

## Publisher's Note

All claims expressed in this article are solely those of the authors and do not necessarily represent those of their affiliated organizations, or those of the publisher, the editors and the reviewers. Any product that may be evaluated in this article, or claim that may be made by its manufacturer, is not guaranteed or endorsed by the publisher.
